# Gene expression trajectories during male and female reproductive development in balsam poplar (*Populus balsamifera* L.)

**DOI:** 10.1038/s41598-020-64938-w

**Published:** 2020-05-21

**Authors:** Quentin Cronk, Raju Soolanayakanahally, Katharina Bräutigam

**Affiliations:** 1 Department of Botany, University of British Columbia, Vancouver, BC V6T 1Z4 Canada; 2 Indian Head Research Farm, Agriculture and Agri-Food Canada, Indian Head, SK S0G 2K0 Canada; 3 Department of Biology, University of Toronto, Mississauga, ON L5L 1C6 Canada

**Keywords:** Flowering, Plant development, Plant reproduction, Transcriptomics, Developmental biology, Molecular biology, Plant sciences

## Abstract

Plant reproductive development from the first appearance of reproductively committed axes through to floral maturation requires massive and rapid remarshalling of gene expression. In dioecious species such as poplar this is further complicated by divergent male and female developmental programs. We used seven time points in male and female balsam poplar (*Populus balsamifera* L.) buds and catkins representing the full annual flowering cycle, to elucidate the effects of time and sex on gene expression during reproductive development. Time (developmental stage) is dominant in patterning gene expression with the effect of sex nested within this. Here, we find (1) evidence for five successive waves of alterations to the chromatin landscape which may be important in setting the overall reproductive trajectory, regardless of sex. (2) Each individual developmental stage is further characterized by marked sex-differential gene expression. (3) Consistent sexually differentiated gene expression regardless of developmental stage reveal candidates for high-level regulators of sex and include the female-specific poplar *ARR17* homologue. There is also consistent male-biased expression of the MADS-box genes *PISTILLATA* and *APETALA3*. Our work provides insights into expression trajectories shaping reproductive development, its potential underlying mechanisms, and sex-specific translation of the genome information into reproductive structures in balsam poplar.

## Introduction

In most animals, reproductive development is a single process resulting from the unitary development of the organism. However, in long lived plants, such as trees, reproductive development is iterated multiple times from vegetative meristems on an annual basis. The process by which meristems are programmed away from leaf production to the production of floral organs and flowers is a remarkable example of stem cell reprogramming.

In poplars (*Populus* spp.), when new axillary meristems are produced on shoots emerging after spring bud break, these meristems are at first uncommitted, but quickly take on a vegetative or reproductive identity^1,2^. In both cases the meristems first produce protective bud scales on their flanks. Then, in vegetative buds, there follows the production of numerous preformed leaf primordia, which will not expand until the following spring (after bud break). Axillary meristems committed to reproduction, on the other hand, do not produce leaf primordia but they form a domed meristem (inflorescence primordium), which in turn produces floral primordia (and their subtending bract scales) on its flanks. These immature inflorescences can be detected inside axillary buds in May or June (depending on seasonal timing). The floral primordia then undergo floral organogenesis before going into winter dormancy. In the spring, sporogenesis occurs inside stamens and carpels and finally floral maturation, flowering and gametophyte production. Each stage of this process requires a different developmental program. In rapid succession there needs to be a massive and timely remarshalling of gene expression, in successive and overlapping waves. *Populus* is a promising model for floral development as its genome is sequenced (from a female tree) and because of its status as a model tree^3,4^.

As poplar is a dioecious tree, this remarkable developmental trajectory is further complicated by sexual dimorphism. There are two substantially different developmental trajectories dependent on individual gender. Balsam poplar (*Populus balsamifera* L.) has an XY system of sex determination with a small sex determining region (SDR)^5^. Strikingly, one gene, *PbRR9* (the poplar homologue of *Arabidopsis* Response Regulator 17, *ARR17*), is differentially methylated according to sex. It has previously been noted that this gene ‘is potentially a master regulator of poplar gender’^6^. For convenience, in this paper we refer to this gene in poplar as ‘*ARR17*’. *ARR17* has been found to be predominantly expressed in reproductive tissues in poplar^7^. Recently partial repeats of this gene (a *pseudo-ARR17* module) were identified at the SDR, and these inverted repeats negatively regulate *ARR17* in males via DNA methylation, confirming ARR17’s role as master regulator of sex in poplar^6,8^.

Whole transcriptome shotgun sequencing (RNAseq) is a powerful technique for studying developmental changes during the life of an organism, based on changes in underlying gene expression during development^9,10^. RNAseq has previously been employed to study gene expression in floral tissue of Chinese white poplar (*Populus tomentosa* Carrière) but on males only^11^, and in *P. balsamifera*, but at a single time point^12^. We wished to extend this work to study the full reproductive trajectory in both sexes. In this study we are particularly concerned with (a) studying temporal expression of genes involved in chromatin modification and (b) elucidating the temporal dimension of sex-specific genes. Late expressed genes may be restricted in expression to a single sex merely as downstream consequences of sexual development: because they are involved in stamen or carpel development and these organs only occur in a single sex. On the other hand, genes with early sexually differentiated expression, or consistent sexually differentiated gene expression regardless of developmental stage, are candidates as higher-level regulators of sex. A developmental series combined with RNAseq is therefore of considerable potential value.

## Results

### Phenotypic trajectory of reproductive development

**Figure 1:**
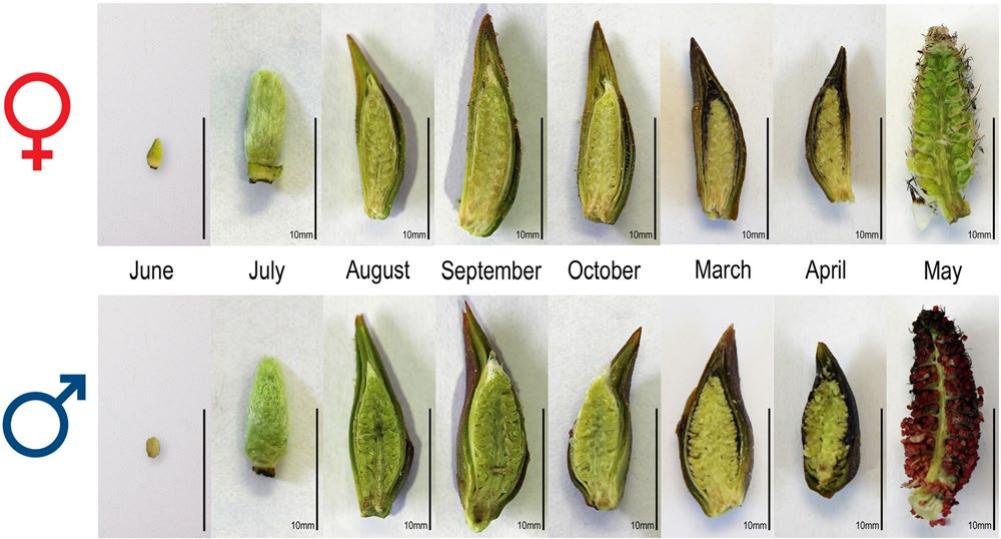
Female (top row) and male (bottom row) reproductive inflorescence development over the course of the year in *Populus balsamifera*. Scale bar: 10 mm.

Most of the floral organogenesis occurs in the summer particularly in the months July to September for the production of mature catkins in the coming year (Fig. 1, Table 1). July and August are the warmest months in the collection locality with average daytime high temperatures around 25 °C. By the end of October, the floral organs are all present although not fully mature. We did not collect during the dormant period November through February as temperatures are consistently below zero during this period and frequently below −20 °C. Our March collection showed the flowers beginning to enlarge as growth resumes, but they were still not fully mature. Our final time point (early May) revealed fully mature pre-anthesis catkins. The short growing season of southern Saskatchewan means that the developmental trajectory of *P. balsamifera* over the summer is compressed, and spring flowering delayed until ecodormancy is broken with the accumulation of heat units.

**Table 1:** Developmental stages sampled of balsam poplar trees at Indian Head, Saskatchewan, Canada.

Date sampled	Length of inflorescence bud (mm, bud scales removed)	Phenotypic developmental markers (female)	Phenotypic developmental markers (male)
21 June	2	Domed inflorescence meristem, no obvious floral primordia visible, No visible difference between male and female inflorescence buds	
21 July	4	Floral primordia as raised bumps present over surface, No visible difference between male and female inflorescence buds	
21 August	6	Slight difference from male in shape of the floral primordium (absence of stamen whorl)	Floral primordia well developed, some indication of floral organ development
21 Sept.	7	No stamen primordia visible, gynoecium primordium visible	Stamen primordia visible in most flowers
21 Oct.	8	Carpels well developed but small	Stamens well developed but small
21 March	10	Some enlargement of all floral organs	Some enlargement of all floral organs
2 May	15	Pre-anthesis female flowers fully developed	Pre-anthesis male flowers fully developed

Sampling occurred in 2017 (months 6–10) and 2018 (months 3 and 5).

### Global patterns of gene expression relative to time and sex

**Figure 2:**
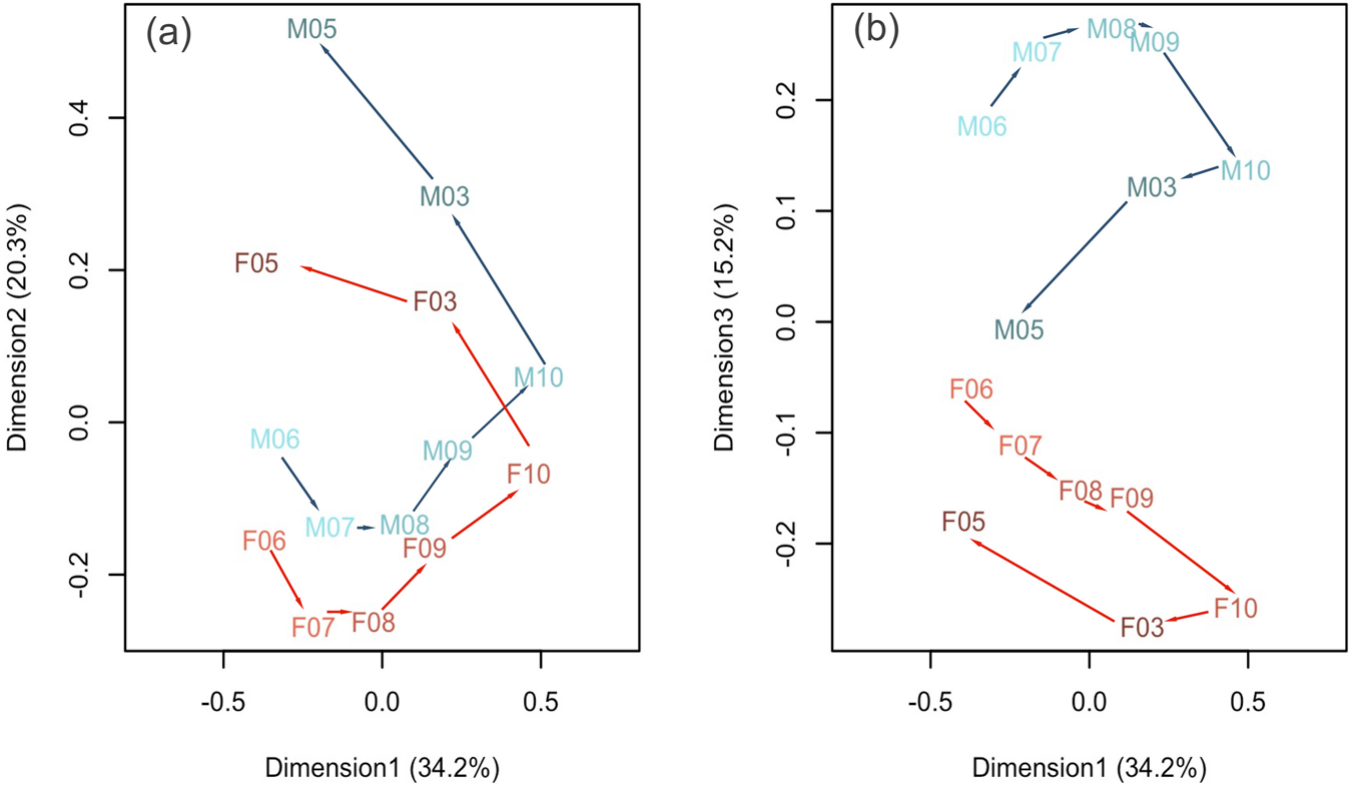
Ordination of gene expression showing developmental trajectory (arrows) and gender (colour: blue = male; red = female). Axes 1 & 2 of the MDS represent variation with time (A). Axis 3 represents variation by gender (B). Axes 1 & 2 together (time) account for 54.5% of the variation. Axis 3 (sex) accounts for 15.2% of the variation. Sample labels are as follows: F/M represents female or male, and numbers correspond to months. E.g. F06 corresponds to the female buds harvested in June.

**Figure 3:**
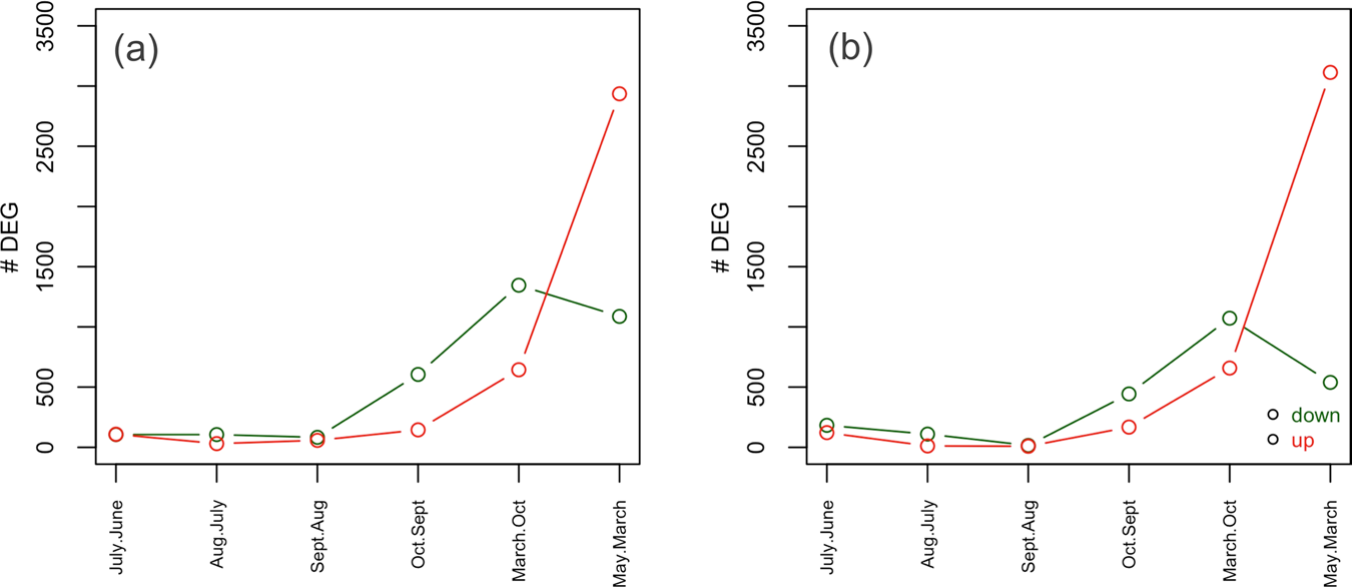
Rate of gene expression change between successive sampling points increases over time in female (a) and male (b) reproductive buds. Red is upregulation over time, green is downregulation. Little month-to-month change in expression occurs over the first few time points, but this rapidly increases as more complex organs develop which have gene expression that is specific to particular developmental time point. The final time point has a large amount of specific gene expression associated with reproductive organ and pollen formation. DEG: differentially expressed genes.

When gene expression patterns are taken as a whole, unsurprisingly time is the dominant explanatory factor and sex is nested within time. The ordination (Fig. 2a) shows that axis one separates time points but not sex. Axis two separates time points with some slight separation of sexes. However, axis three separates sexes strongly and clearly (Fig. 2b). Axes 1 and 2 account for 54.5% of the variance and axis 3 accounts for 15.2%. We next looked at differential expression between adjacent time points regardless of the sex of the reproductive bud (Fig. S1). Very similar patterns are also obtained when female and male buds are considered separately (Fig. 3). Rather surprising, when comparing early monthly transitions (June to July, July to August and August to September) the absolute number of genes being activated or repressed is small even though the tissues at this stage are highly developmentally active. There is a jump in the September to October comparison and this may be due partly to the greater complexity of the structures, and partly to preparation for endodormancy. The October to March comparison is even more striking perhaps due to breaking of ecodormancy and resumption of growth within the bud. Finally, the most dramatic transcriptomic reprogramming occurs between March and May. This may be due in part to the activation of the large pollen transcriptome. GO categories for pollen wall assembly, pollen exine formation and sporopollenin biosynthetic process are over-represented at this time point (Table 2).

**Table 2:** Over-represented GO categories among genes with time-dependent sex differences in expression.

GO Category	6 (June)	7 (July)	8 (Aug)	9 (Sept)	10 (Oct)	3 (Mar)	5 (May)
GO:0048367 shoot system development		M	M	F	M	M	F
GO:0090567 reproductive shoot system development		M	M	M, F	M	M	F
GO:0009908 flower development		M	M	M, F	M	M	F
GO:0048438 floral whorl development		M	M	M, F		M	M, F
GO:0048437 floral organ development		M		M, F	M	M	F
GO:0048449 floral organ formation				F			
GO:0048467 gynoecium development				F			
GO:0090691 formation of plant organ boundary				F	F	F	
GO:0048443 stamen development						M	M,F
GO:0048653 anther development						M	M
GO:0048658 anther wall tapetum development						M	M
GO:0010208 pollen wall assembly							M
GO:0010584 pollen exine formation							M
GO:0080110 sporopollenin biosynthetic process							M

For each individual time point, GO categories that are enriched in genes with significant expression difference between male and female samples are summarized. Only categories related to development are shown here as they represent a major proportion of significantly enriched GO categories. M and F mark categories which are enriched in genes with male or female biased expression, respectively. Difference in transcript abundance between the sexes was calculated independently for each time point, i.e. there can be overlaps in the gene sets between time points. See also Fig. 7: Venn diagram. A full list of enriched GO categories and their associated P-values (g:SCS algorithm for multiple testing correction) is given in Table S3.

Generally, the acceleration of transcriptomic reprogramming between time points appears to reflect a combination of environmental adaptation (moving to and from winter) and the greater complexity and physiological activity of the organs themselves as they move towards maturity.

### A time series of chromatin alteration

**Figure 4:**
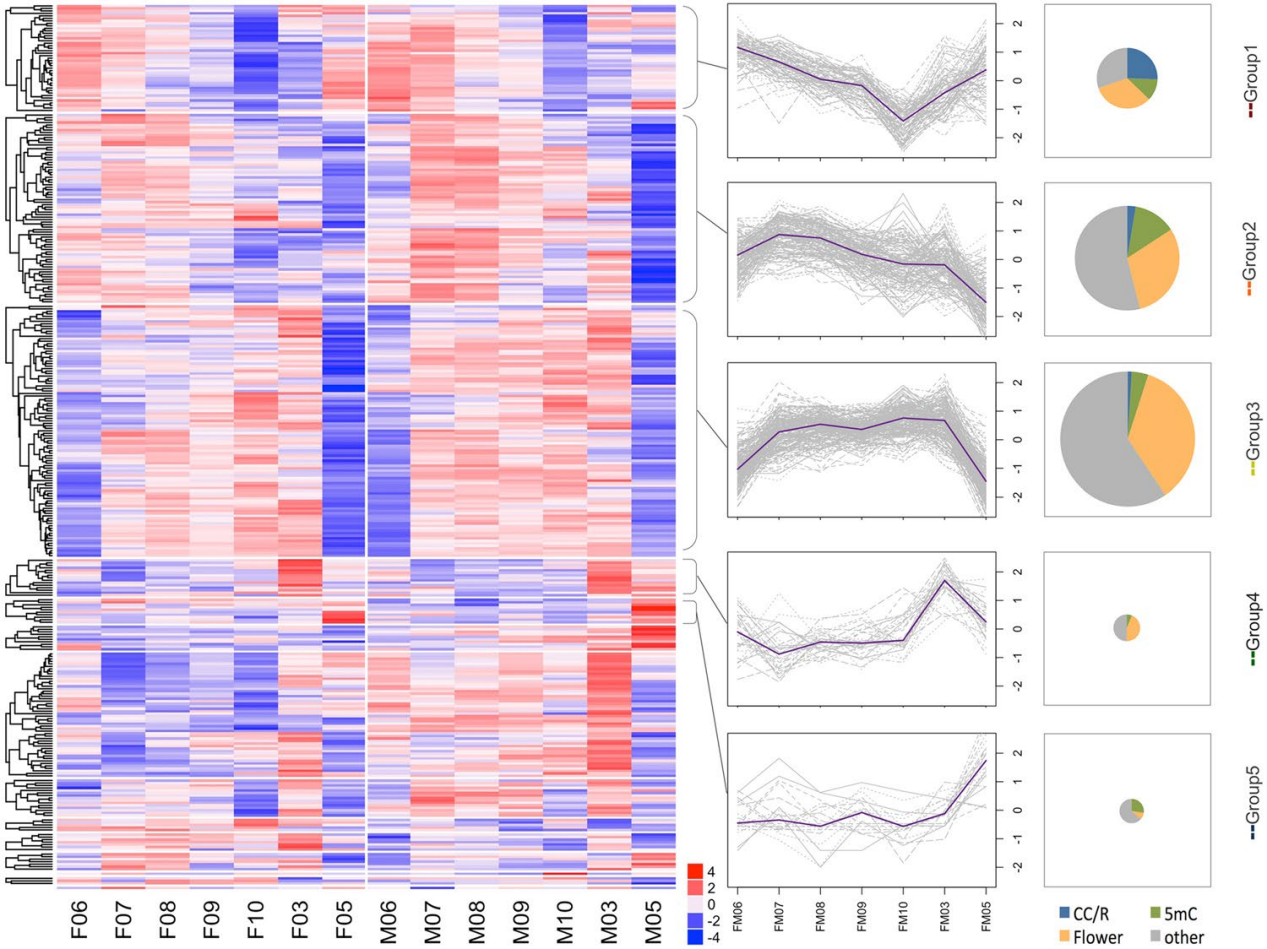
Groups of chromatin modification genes show strong temporal patterns (heat map). Transcript abundance data for all expressed poplar homologs of genes in the functional category ‘covalent chromatin modification’ (GO:0016569, AMIGO db, *Arabidopsis*) are shown. Data were subjected to hierarchical clustering, subdivided based on major clusters, and also plotted as line graphs to visualize temporal change. Additional functional information was retrieved from recent publications and is shown as pie charts. Group1 includes DNA methylation and cell-cycle related compounds (*CMT3, DDM1, KYP*), histone methyltransferases (*JMJ12/REF6, JMJ13*, regulation of flowering); Group 2: RdDM (*AGO4, DRD1, SHH1), FLD, trxG* (*ATX1*, floral organ, flowering); Group 3: histone methyltransferases (*JMJ16, JMJ20, ASHH2*, flowering, organ development); Group 4: ubiquitination (*UBC2, ENY*), histone deacetylase (*HDA19*, flowering, floral patterning). Some chromatin modification genes show male bias (*PRMT3,5*, and *6*). Interestingly very little chromatin modification activity is evident at the final time-point (mature buds, just prior to anthesis). Line plots show median (purple line) and individual (grey) expression patterns per group. The size of the pie charts reflects the number of genes in each respective group, additional biological functions curated from recent literature are color coded. CC/R: cell cycle or DNA repair, 5mC: DNA methylation at cytosine, Flower: role in the regulation of flowering, flowering time or flower organ development. Sample labels: F/M represents female or male; numbers correspond to months. E.g. F06 corresponds to female buds harvested in June.

Among the classes of genes that could be looked at in detail for temporal patterns, we were particularly interested in the genes associated with chromatin modification (GO:0016569). Chromatin modification has important consequences for gene expression and development and might be thought of as the “tramlines” along which a developmental trajectory could run. Broadly speaking we can divide chromatin modification associated genes into five major time-dependent categories with early, early middle, middle-late, late and very late regulation (see the heat map and line plots for groups 1 to 5 in Fig. 4, Table S1). In the following report of particular genes, *Arabidopsis* gene names are used but the corresponding *Populus* gene i.d. numbers are given in Table 3.

**Table 3:** *Populus* genome (v.3) identifiers for main genes mentioned in text.

Abbreviation	*Arabidopsis* gene name	*Populus* gene i.d.
*ADA2*	*TRANSCRIPTIONAL ADAPTER 2*	Potri.004G135400; Potri.016G007600
*AGO* type	*ARGONAUTE* type	Potri.001G219700
*AGO4*	*ARGONAUTE 4*	Potri.006G025900
*AHK4*	*HISTIDINE KINASE 4*	Potri.010G102900; Potri.008G137900
*AHP5*	*HISTIDINE PHOSPHOTRANSFER PROTEIN 5*	Potri.014G136200
*AP3*	*APETALA 3*	Potri.005G118000; Potri.007G017000
*ARR9,17,22*	*ARABIDOPSIS RESPONSE REGULATOR*	Potri.006G041100; Potri.019G133600; Potri.001G050900
*ASHH2*	*ASH1 HOMOLOG 2*	Potri.002G079100
*ATX1*	*TRITHORAX RELATED 1*	Potri.018G023000
*ATXR6*	*TRITHORAX RELATED 6*	Potri.012G018300; Potri.015G009600
*AUR1,3*	*AURORA KINASE*	Potri.006G235000; Potri.006G080500
*BRCA1*	*BREAST CANCER SUSCEPTIBILITY 1*	Potri.001G358100
*CDKB2;1*	*CYCLIN-DEPENDENT KINASE B2;*1	Potri.002G003400
*CHR11*	*CHROMATIN REMODELLING 11*	Potri.010G021400; Potri.010G019800
*CLC-C*	*CHLORIDE CHANNEL-C*	Potri.018G138100
*CLF*	*CURLY LEAF*	Potri.005G140200
*CMT3*	*CHROMOMETHYLASE 3*	Potri.001G009600
*CRF5*	*CYTOKININ RESPONSE FACTOR 5*	Potri.014G094500; Potri.001G094800
*CYP71*	*CYCLOPHILIN 71*	Potri.004G186000
*DDM1*	*DECREASED IN DNA METHYLATION*	Potri.007G026700; Potri.019G129900; Potri.T120000
*DRD1*	*DEFECTIVE IN RNA-DIRECTED DNA METHYLATION 1*	Potri.004G159000
*ENY2*	*ENHANCER OF YELLOW 2*	Potri.T061800
*FLD*	*FL0WERING LOCUS D*	Potri.010G227200
*GATA21*	*GATA TRANSCRIPTION FACTOR 21*	Potri.006G229200
*GCN5*	*HISTONE ACETYLTRANSFERASE GCN 5*	Potri.002G045900; Potri.005G217400
*HDA6,19*	*HISTONE DEACETYLASE*	Potri.012G083800; Potri.009G170700
*JMJ13,16,17,20,27*	*JUMONJI DOMAIN-CONTAINING PROTEIN*	Potri.001G137700; Potri.019G018700; Potri.003G126600; Potri.015G080400; Potri.015G012800
*KYP*	*KRYPTONITE*	Potri.003G162800; Potri.014G143900
*PI*	*PISTILLATA*	Potri.005G182200; Potri.002G079000
*PRMT3,5,6*	*PROTEIN ARGININE N-METHYLTRANSFERASE*	Potri.001G030200; Potri.018G000500; Potri.007G000300
*REF6 / JMJ12*	*RELATIVE OF EARLY FLOWERING 6 (JMJ 12)*	Potri.012G092400
*SAHH1*	*S-ADENOSYL-L-HOMOCYSTEINE HYDROLASE 1*	Potri.001G320500
*SHH1*	*SAWADEE HOMEODOMAIN HOMOLOG 1*	Potri.001G189200
*STK*	*SEEDSTICK*	Potri.013G104900; Potri.019G077200
*SUVH5*	*SUPPRESSOR OF VARIEGATION 3–9 HOMOLOG PROTEIN 5*	Potri.015G111900; Potri.012G115500
*SUVR4*	*SUPPRESSOR OF VARIEGATION 3–9-RELATED PROTEIN 4*	Potri.013G048100
*SVP*	*SHORT VEGETATIVE PHASE*	Potri.005G155700
*TAF14B*	*TRANSCRIPTION INITIATION FACTOR TFIID SUBUNIT 14B*	Potri.007G012700
*TCP-1*	*T-COMPLEX PROTEIN 1 SUBUNIT ALPHA*	Potri.018G138200
*UBC2*	*UBIQUITIN-CONJUGATING ENZYME 2*	Potri.013G064400; Potri.015G063900
*VRN5*	*VERNALIZATION 5*	Potri.018G076500

Of the genes showing strong temporal patterns, group 1 (early and partially very late) includes homologs of the following *Arabidopsis* genes: *DECREASED IN DNA METHYLATION 1* (*DDM1*) encoding a chromatin remodeler, the histone methyltransferase *KRYPTONITE* (*KYP*), an argonaute gene (Potri.001G219700) and a homologue of the DNA methyltransferase *CHROMOMETHYLASE 3* (*CMT3*), i.e. genes with functions in DNA methylation (Table S1). Group 1 is also characterized by type B cyclin-dependent kinase (*CDKB2;1*), aurora kinases (*AUR1, 3*), a *BRCA1* homologue, and *ARABIDOPSIS TRITHORAX-RELATED PROTEIN* 6 (*ATXR*6), all of which with functions in cell cycle and/or DNA repair. Finally JmjC containing histone demethylases (*JMJ12/RELATIVE OF EARLY FLOWERING 6* (*REF6*)*, JMJ13*), and polycomb repressive complex 2 (PRC2) genes *CURLY LEAF* (*CLF*) and *VERNALIZATION 5* (*VRN5*) share patterns of early regulation.

Similarly, group 2 with early-mid regulation is characterized by homologs to the *Arabidopsis* DNA methylation pathway components encoded by *DEFECTIVE IN RNA-DIRECTED DNA METHYLATION 1* (*DRD1*), *AGO4* (Potri.006G025900), and *SAWADEE HOMEODOMAIN HOMOLOG 1* (*SHH1*). Most genes in group 2 code, however, for generic histone modifiers or histone modifying enzymes for which functions in the regulation of flowering have been described. The latter include homologs to the *Arabidopsis* histone demethylase *JMJ27*, transcription initiation factor *TAF14B*, trithorax-related histone methyltransferase *ATX1* and *FLOWERING LOCUS D* (*FLD*).

Group 3 with mid-late regulation comprises predominantly genes coding for generic and flowering-related histone modifiers as well as genes related to organ formation. Homologs to the *Arabidopsis* WD40 Cyclophilin 71 (CYP71), JMJ 16, 17 and 20 and histone deacetylase HDA6 fall into this category. Group 4 (late) contains genes annotated to histone acetylation (*HISTONE DEACETYLASE 19 HDA19*) and histone (de)ubiquitation (*ENY2* and *UBC2*). Very little chromatin modification is evident at the final time-point (mature buds, prior to anthesis) with consistent patterns among males and females. The exception is group 5, a small group of genes coding for a variety of different functions (including homologs to *Arabidopsis SUVH5* and *SAHH1*: Potri.015G111900, Potri.001G320500).

Not all genes in the chromatin modification pathway show temporal regulation, but instead show gender-based patterns. Some chromatin modification genes show male bias (Fig. 4: below group 5). These include, homologs of the *Arabidopsis* histone arginine methyltransferases *PRMT3,5* and *6*, the transcriptional adapter ADA2, and a SET domain containing histone methyltransferases (*SUVR4*).

**Figure 5:**
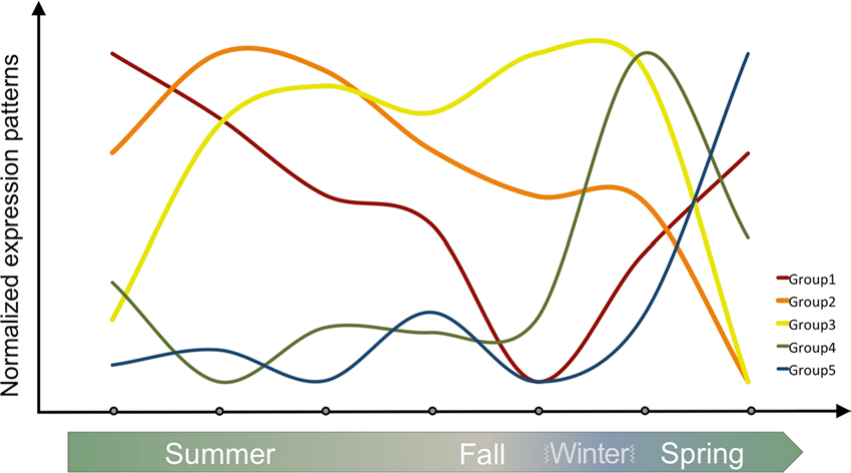
Gene expression patterns indicate a time series of chromatin organization events. This schematic serves as an explanatory diagram for the patterns observed in the heat map (see Fig. 4 for details). The thickness of the line reflects the number of genes associated with each of the major groups highlighted in Fig. 4. Note that little chromatin modification activity is evident at the final time-point (mature buds, just prior to anthesis).

To highlight the successive expression waves of genes related to covalent chromatin modification throughout reproductive development, median expression of gene groups 1–5 are shown in Fig. 5.

### Time-dependent sex-specific gene expression

**Figure 6:**
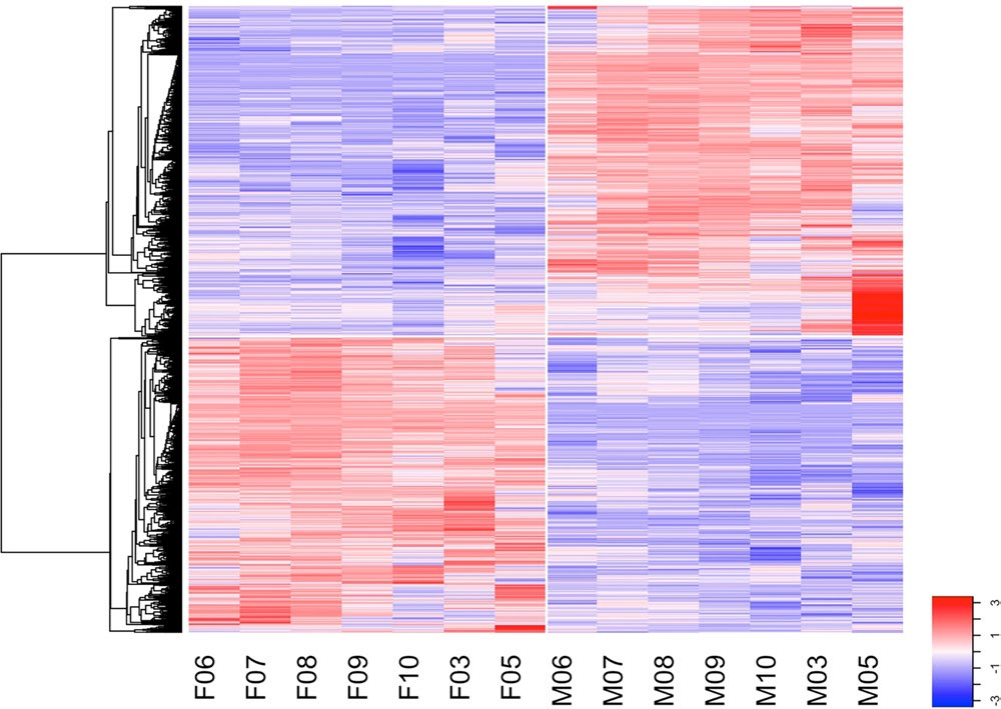
Heat map of overall sex differentially expressed genes (high expression red, low expression blue) as determined by comparing all female samples to all male samples regardless of time point (main effect of sex). Some genes have high expression in females (F samples), while other have high expression in males (M samples). Some genes have highly consistent patterns of gene expression at all time-points, while others show some more variability. For a subset of genes, the very strong expression at the final time point (bright red block, mature male buds, May) drives the contrast. These genes are associated with pollen. Sample labels: F/M represents female or male; numbers correspond to months. E.g. F06 corresponds to female buds harvested in June.

Plant sex has a very strong effect on gene expression patterns right through reproductive development, even at the earliest stages when no obvious morphological differences are evident between males and females. Figure 6 shows a heat map of the significantly differentiated genes between genders by comparing all female samples against all male samples (main effect of sex, Table S2). This identified 1195 genes with a significant male and 1071 genes with a significant female bias. It can be seen that some genes have consistent patterns of gene expression across all months (time-independent sex differentiation). These will be discussed in the next section. Others have patterns of differential sex-specific expression that is most marked at particular time-points (time-dependent sex differentiation). For instance, a group of genes have very strong expression in males (bright red block) at the final time point in May (M05). These include a number of genes associated with pollen. The GO analysis (Table 2) shows that genes expressed in May are over-represented for categories including: anther development, pollen wall assembly, pollen exine formation and sporopollenin biosynthetic process.

**Figure 7:**
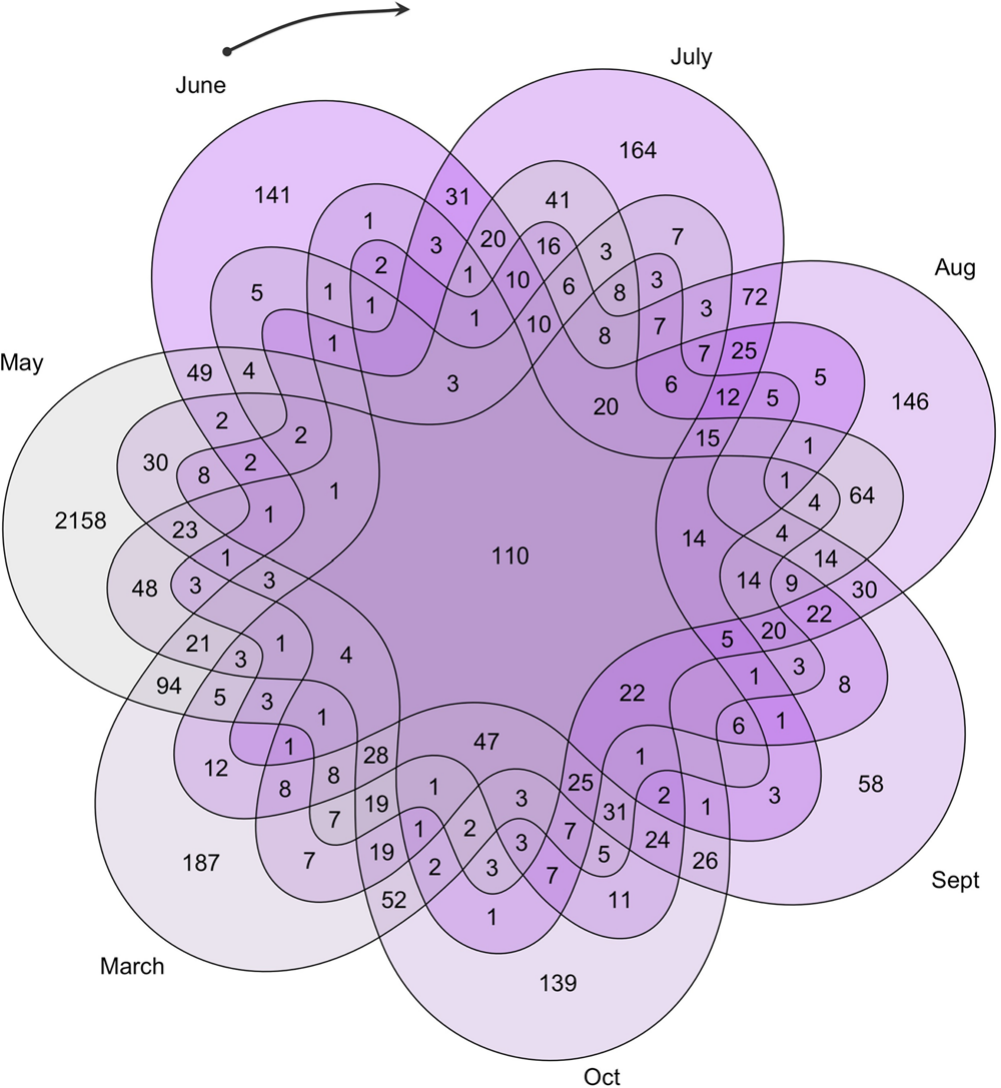
Venn diagram of sex differentially expressed genes based on independent comparisons for each month. Some genes (110, middle) have differential expression between sexes in all the seven time points (time independent). Other genes (outer numbers) have significant differential expression only in a single month (time-dependent). All other fields indicate overlaps in sex differential expression for all possible combinations of two or more time points. The black arrow marks the beginning of the annual flowering cycle, with June representing the earliest developmental stage studied. By our final time point, May, fully mature pre-anthesis catkins have developed (c.f. Figure 1 for images of reproductive inflorescences at the different time points.).

The Venn diagram (Fig. 7) gives a month-by-month analysis. Certain genes are sex differentially regulated in all months (central number, 110) but the largest categories are generally of those genes sex differentially regulated only in a single month which may indicate a specific developmental process. The GO analysis (Table 2, Tables S3, S4) gives some insight into this and gives an idea of the underlying molecular basis of the developmental trajectory. Very striking is the large number of sex differentially regulated genes associated with the last month, May. This is the month in which pollen is being produced, as the GO analysis indicates.

### Time-independent sex-specific gene expression

An interesting category is those genes that are consistently sex regulated at all (or nearly all) stages of reproductive development. This could imply sex regulation at a high level, for instance by the response regulator, poplar homologue of ARR17, which is the gene now known to function as the master regulator of sex^6,8^. Poplar ARR17 (Potri.019G133600) is consistently female-specific at all stages except for the latest time point. Sex-specific expression of ARR17 is unsurprising as this gene is directly regulated by a factor at the sex determining region (SDR)^8^, resulting in heavy DNA methylation of this gene in males but not in females^6^. However, other response regulators, the poplar homologues of ARR22 and ARR9 (Potri.001G050900, Potri.006G041100), not located at the sex locus, have a consistent male-biased pattern, perhaps indicative of a negative interaction with ARR17, although ARR22 is very weakly expressed.

Other genes with consistent male bias include two homologs of *Arabidopsis PISTILLATA* and two homologs of *Arabidopsis APETALA3* (*AP3*). A gene with consistent female bias (except in the final, mature bud stage) is one homolog of *Arabidopsis SVP* (Potri.005G155700) and two homologs of *Arabidopsis SEEDSTICK* (*STK*).

The identical (male) pattern of expression of *PI* and *AP3* in our data is not surprising as their gene products form a heterodimer, and together (in concert with other genes) they regulate petal and stamen development in *Arabidopsis*. More surprising is the consistent and uniform sex-specific expression of these genes throughout reproductive development in poplar which might imply that they are under general sex-specific control. In *Arabidopsis*, these genes are positively regulated by LFY and AP1 (in concert with UFO) and negatively regulated by EMF1 & 2^13,14^. PI/AP3 is a negative regulator of *GATA21/GNC* in *Arabidopsis*^15^ and consistent with this, one copy of this gene in our poplar data (*GATA21*) is female specific.

Further genes with consistent male bias include cytokinin receptor homologs of *Arabidopsis AHK4*, *AHP5*, which is responsible for propagating a cytokinin signal from receptors to B-type response regulators, or the *CYTOKININ RESPONSE FACTOR 5* (*CRF5*: Potri.014G094500) which is upregulated by B-type response regulators.

Among chromatin modifying genes, two homologs of an *Arabidopsis* chromatin remodeling ATPase *CHR11*, show, interestingly, either male (Potri.010G021400) or female (Potri.010G019800) bias. Other consistently male biased genes are a homolog of *SUVH5* (Potri.012G115500) and of the transcriptional adapter *ADA2* (Potri.004G135400), involved in histone acetylation and cytokinin signal mediation. The latter shows an interesting anti-correlation in expression to those of a *CRF5* homolog (Potri.001G094800), which is also male-biased. Genes that have consistent female specificity are a chromatin-remodeling complex subunit gene (Potri.T092100), and a methyl-DNA-binding domain genes (Potri.018G146700).

### Sex-biased gene expression at the sex determining region (SDR)

**Figure 8:**
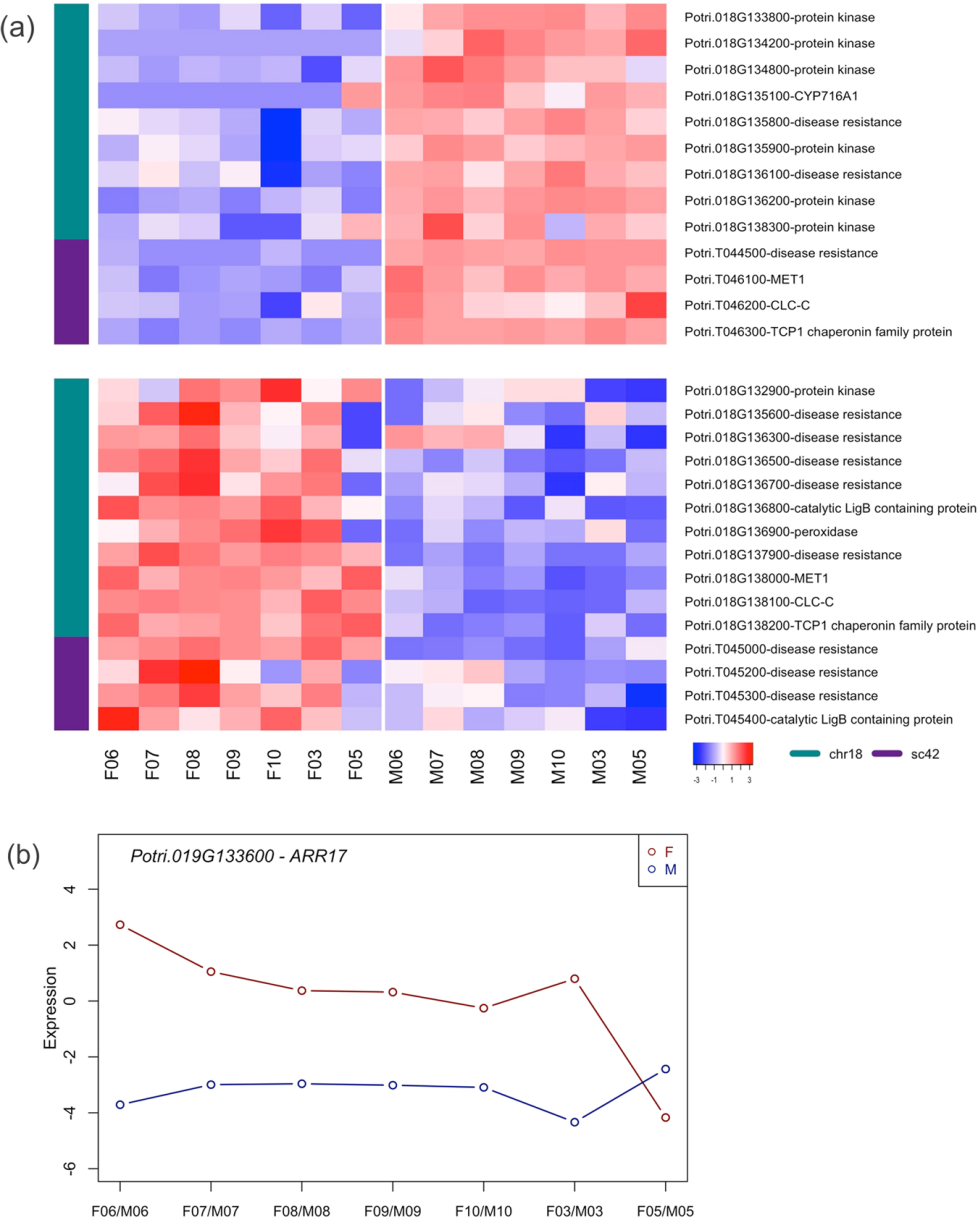
Genes at or near the sex determining region are differentially expressed by gender. (A) Direction of differential expression (top panel – male biased or bottom panel - female biased) is strongly dependent on the spatial organization of the genes (see Table 4). This heat map includes genes from chromosome 18 and unassigned contig scaffold 42 (the sex determining region is actually on chromosome 19 but it is misassembled in v.3 of the *Populus trichocarpa* genome^5^). Genes with significant differential regulation are detailed in Table 4. (B) Expression of putative sex determining (ferminizing factor) gene *ARR17* (gene with female-specific expression assembled to chromosome 19, see text.) The strongest sex-differential expression is at the earliest stage. The y-axis represents expression intensity as log cpms (counts per million). Sample labels: F/M represents female or male; numbers correspond to months. E.g. F06 corresponds to female buds harvested in June.

The sex determining region on chromosome 19 is incorrectly assembled to chromosome 18 (and other regions including scaffold 42) in the poplar genome v.3, but the order of the genes is consistent^5^. One thing is evident: there is a striking sex-specific expression of several of these genes (Fig. 8; Table 4).

**Table 4:** Genes with significant differential expression at or near (or associated with) the SDR (see also Fig. 8) across all time points.

Gene class	Gense number (order)	Expression bias
*Chromosome Potri.018*		
protein kinase superfamily protein	133800	M
protein kinase superfamily protein	134200	M
protein kinase superfamily protein	134800	M
disease resistance gene^$^	135800	M
disease resistance gene	135900	M
disease resistance gene^$^	136100	M
disease resistance gene	136200	M
disease resistance gene^$^	136500	F
catalytic LigB containing protein	136800	F
peroxidase superfamily^$^	136900	F
disease resistance gene	137900	F
**methyltransferase 1 (MET1)**	**138000**	**F**
**chloride channel C (CLC-C)** ^$^	**138100**	**F**
**chaperonin family protein (TCP1)** ^$^	**138200**	**F**
*Scaffold 42*		
disease resistance gene	044500	M
disease resistance gene	045000	F
disease resistance gene	045300	F
**methyltransferase 1 (MET1)**	**046100**	**M**
**chloride channel C (CLC-C)**	**046200**	**M**
**chaperonin family protein (TCP1)**	**046300**	**M**
*Chromosome Potri.019*		
homolog of ARR17	133600	F

Although these genes are not all assembled together in v.3 of the *Populus trichocarpa* genome they are all at or near the SDR (see text). Blocks of similar expression polarity can readily be seen. Bold marks three genes (MET1, CLC-C and TCP1) which appear to be duplicated but have reversed expression polarity. ^$^similar expression trends were observed in data from Sanderson *et al*., 2018 at one individual time point late in flower development^12^.

Genes at or near the SDR with consistent male bias include the following (Table 4): protein kinase superfamily proteins (Potri.018G133800/ 134200/ 134800); disease resistance genes (Potri.018G135800/ 135900/ 136100/ 136200 and Potri.T044500); methyltransferase 1 (Potri.T046100); chloride channel C (Potri.T046200); and chaperonin family protein (Potri.T046300).

Significantly female biased genes at (or directly regulated by) the SDR include the following (Table 4): homolog of ARR17 (Potri.019G133600); disease resistance genes (Potri.18G136500/137900 and Т045000); catalytic LigB containing protein (Роtri.018G136800); peroxidase superfamily (Роtri.018G136900); methyltransferase 1 (Роtri.018G138000); chloride channel C (Роtri.018G138100) and chaperonin family protein (Роtri.018G138200).

The male versus female expression polarity at the SDR does not appear to be spatially random but occurs in blocks (Table 4). This could be due to epigenetic patterning at the SDR (perhaps chromatin status) and indicate that there may be larger-scale epigenetic patterning present at the SDR. A group of three genes (*MET1*, *CLC-C* and *TCP1*) appears to be duplicated (assembled to chromosome 18 and scaffold 42) yet the duplicates have opposite expression polarity. An X versus Y allelic copy can be ruled out as the *P. trichocarpa* genome was assembled from an XX female. However, it should be borne in mind that the region near the SDR has problematic assembly^5^. The presence of members of large genes families (e.g. disease resistance and protein kinase gene families) could result in mapping problems and the distinction between duplicated blocks and haplotypes within this region could be problematic^16^.

## Discussion

### The time course of development in relation to chromatin modification

Interpretation of information encoded in the genome has to be tightly controlled to ensure correct spatial and temporal development and proper organ function. In this process, chromatin organization can play a central role. Chromatin structure is influenced by chemical modifications to DNA, post-translational modification to histone tails or by remodelers, and it can shape gene expression patterns at individual genetic loci as well as larger genomic regions. In the following discussion, *Arabidopsis* gene names are used but the corresponding *Populus* gene i.d. numbers are given in Table 3.

In our data set, genes assigned to the functional category “covalent chromatin modifications” fall into five major groups with consecutive expression peaks throughout reproductive development in balsam poplar (Figs. 4 and 5). Genes with early expression peaks are specifically characterized by functions related to cell cycle, DNA repair mechanisms (group1, Figs. 4 and 5), and DNA methylation pathways (groups 1 and 2). They include, homologs of *Arabidopsis* aurora kinases (*AUR1, 3*) involved in histone phosphorylation, *BRCA1* with DNA repair functions, a cyclin-dependent kinase (*CDKB2;1*), subunits of histone acetyltransferase complex NuA4 (Potri.002G038700, Potri.009G160600, Potri.014G000600, Potri.014G117400), or RdDM components (*DDM1, AGO4, DRD1, SHH1*). This likely reflects enhanced cell division activities that occur early in floral reproductive development and the need to maintain or modify DNA methylation patterns during enhanced cell division.

Genes with early-to-late expression peaks (groups 1 to 4, Figs. 3 and 4) contain a remarkable proportion (roughly 1/3) whose *Arabidopsis* counterparts have been implicated in the regulation of flowering, flowering time, and floral organ development. Genes with an early expression peak include examples for photoperiodic regulation of flowering time (*JMJ13* homolog) or organ boundary formation (*REF6*)^17–20^. Intermediate expression peaks (groups 2 and 3) were detected for homologs of well-described *Arabidopsis* genes such as *GCN5* with a role in inflorescence meristem size regulation^21^, *FLD*, which is also involved in the activation of floral meristem identity genes^22–24^, *ATX1*, which is required to correct expression of floral homeotic genes and thus flower organ identity^25^, or *ASHH2* which functions in the regulation of flowering time and is critical for ovule and anther development^26,27^. This is followed by late expression peaks in, for example, Potri.009G170700, for which the *Arabidopsis* counterpart *HDA19* fulfils multiple roles ranging from timing of flowering to regulation of floral patterning^28^.

At mechanistic level, the trithorax group (trxG) protein ATX1, for example, serves as histone methyltranserase that sets trimethylation at histone 3 lysine 4 (H3K4me3). It is involved in maintaining the active state of various floral homeotic genes early in development^25^. These marks can antagonize the action of CLF (group1), a polycomb group (PcG) protein that catalyzes H3K27me3 indicative of repressive chromatin states^25^. Recently ATX1 and CLF have also been reported to create chromatin with both, activating and repressive marks at the floral homeotic locus AGAMOUS (AG) in leaves. Such silent bivalent chromatin is thought to be poised for transcription at a later time point^29^. This concept might open up interesting possibilities for a fast and flexible integration of various signals into the regulation of flowering. Many of the components involved in chromatin regulation act as part of protein complexes. The histone deacetylase HDA5 was shown to interact with HDA6 and FLD^23^. HDA19 and HDA6 complexes share components, while maintaining distinct functions^30^, and ATX1 is thought to function in a COMPASS-like complex^31^.

How chromatin modifying enzymes are targeted to specific loci in the genome is a matter of ongoing research. Affinity purification combined with mass spectrometry identified a direct interactions of the H3K27 demethylase REF6 with the MADS domain proteins AP1 and AG pointing towards a recruitment of REF6 by transcription factors^17^. Further work by Pajoro *et al*., (2014) showed that the MADS domain transcription factors AP1/SEP3 were able to bind target DNA even before the chromatin at target loci was more accessible^32^, supporting this conclusion. At the same time, REF6 was also shown to bind to specific DNA motifs itself through its zinc-finger domain indicating alternative modes of genome targeting by chromatin modifiers^18^.

The above discussed examples rely on work using the model plant *Arabidopsis*. While central to unraveling molecular mechanisms, some fundamental differences to perennials should be considered. *Arabidopsis* flower only once in their life time with one individual meristem transitioning from vegetative to reproductive fate. Commitment to flowering ultimately leads to senescence and the end of the life cycle. To maximize life time reproductive success, the timing of flowering is key. Extensive control of flowering time regulation exists in *Arabidopsis* which, in addition to autonomous and gibberellin pathways integrate vernalization and, importantly, photoperiod (reviewed in e.g. He, 2015)^33^. Perennials such as poplar, on the other hand, flower every spring once maturity is reached and the transition from vegetative to reproductive fate occurs at multiple axillary meristems. While timing of flowering is largely defined by the course of the year in poplar, critical decisions involve which and how many meristems to commit to flowering. In addition, actual flower development needs to be fine-tuned and synchronized with the seasonality. One could also argue that chromatin modifiers with described functions in flowering time regulation in *Arabidopsis* may fulfil related but distinct task in perennials (e.g. determining which meristems to commit to reproduction and/or synchronization of flower development rather than flowering time regulation itself).

In line with this, earlier work indicated parallels between this acquisition of flowering competence in annuals (vernalization, a prime example for integrating external signals into chromatin structure) and bud dormancy cycles in perennials^34,35^. While environmental signals and signaling pathways may overlap, there are, however, indications for functional differences^34,36^.

The diversity of chromatin modifiers, modularity of complexes in which these modifiers function, antagonistic interactions such as those of trxG and PcGs, or bivalent chromatin are molecular mechanisms by which perennials could achieve robust, yet flexible regulation of flowering and flower development. Here, we uncover a time series in the expression of chromatin modifying components in poplar reproductive bud development. This may be indicative of a cascade of events that regulates chromatin organization and function. In future studies, for example by using chromatin immunoprecipitation, it will be interesting to learn how these expression patterns reflect chromatin marks and which signals contribute to chromatin organization in poplar reproductive development.

### Differential gene expression and differential morphology: cause and consequence

In late stage male and female inflorescences, the morphology is already entirely different: pistils in females and stamens in males. Late stage gene expression comparisons are not, therefore, comparing like with like. Late stage differences in gene expression may be due to differences in physiology and development of different organ types: they are the consequences of morphological differences not their cause. This is seen dramatically in our final stage in which a large number of genes with male specific expression is associated with pollen. In contrast, inflorescence primordia of our first stage (June) are morphologically indistinguishable between males and females. Gene expression differences cannot therefore be due solely to the presence of different organs: they are more likely to be causal.

A previous study looked at late stage gene expression in male and female floral tissues in *P. balsamifera* at a single developmental stage sampled at high-latitude in Alaska^12^. This study found 30503 genes expressed in floral tissue, of which 11068 showed sex-biased expression (the majority with female bias). Of the 12 genes located in the genomic SDR, as previously defined^5^, the study found only three to have sex-biased expression one in males (*ARR17*: Potri.019G133600), and two in females (*CLC-C*: Potri.018G138100 and *TCP-1*: Potri.018G138200). This study, with material collected in “early spring”, is broadly comparable to our March (or later) stage. However, we found *ARR17* to be consistently female-biased at all developmental stages except for the latest developmental stage in May when both male and female buds, had negligible levels of *ARR17* expression. It should also be noted that the protein coding version of *ARR17* itself may not be located at the SDR; instead partial repeats of ARR17 are located at the SDR, which function as regulators of *ARR17*^8^.

Concentrating on early-stage differential gene expression (i.e. June and July time points) we found a number of sex-differentially regulated genes. These included *ARR17* which had previously been identified and later confirmed as “master regulator” of sex in poplar^6,8^. Interestingly certain genes associated with flower development were also differentially expressed at this stage, including *PISTILLATA (PI)* and *APETALA3 (AP3)*, which are discussed in the next section.

### Possible mechanisms for the developmental control of male and female flowers in poplar

The strong sexually-specific expression of *PISTILLATA* (*PI*) and *APETALA3* (*AP3*) shown here immediately suggests a mechanism for male and female floral development. *PI/AP3* are absolutely necessary for the formation of stamens so their downregulation would potentially be sufficient to produce female flowers. Conversely, carpels do not form in the presence of PI/AP3. In hermaphrodite flowers *PI/AP3* expression is kept out of the centre of the floral meristem, thus allowing carpels to form. If we hypothesize that *PI/AP3* in balsam poplar is expressed throughout the floral meristem, then no carpels can form and male flowers will result. Normally, *PI/AP3* is kept out of the centre of the floral meristem by regulation via SUPERMAN. Any mutation to compromise this regulation will potentially result in wholly male flowers. If PI/AP3 overexpression is constitutive in poplar, then males would be the “default sex”. A single feminizing gene could then convert to female flowers simply by repressing PI/AP3 expression altogether. In this model a single gene only is required to regulate sex, contrasting with the two-gene model commonly cited^37^.

The *PI/AP3* regulatory hypothesis for dioecy is attractive, but it would be the proximal mechanism for dioecy, not the ultimate cause. Neither PI nor AP3 or any of their immediate regulators are present at the sex-determining region^5^. The SDR harbours, however, partial repeats of *ARR17* (pseudo-*ARR17*) that regulate the primary sex-regulator and feminizing factor ARR17 itself^6,8^. The true protein-coding version of *ARR17* shows sex-differential methylation^6^, likely as a result of repeat-induced RdDM^8^. The question then becomes: can we trace a pathway, however convoluted, from *ARR17* to *PI/AP3*?

*ARR17* as a type-A response regulator would be expected to be a negative regulator of the cytokinin pathway. Expression of *ARR17* leads to females, yet in many species cytokinin treatment has been shown to have a feminizing effect. In *Sapium*, treatment with cytokinin has been shown to lead to a down-regulation of *PI*^38^. In poplar, against expectation, there is male-specific expression of numerous genes in the cytokinin pathway, such as the cytokinin receptor *AHK4*. On the other hand, there is also male-specific (although weak) expression of *ARR22*, a non-canonical A-type (or C-type) response regulator, known to be a negative regulator of B-type RR function, that is necessary for cytokinin signal transduction^39^. There is thus at present no obvious simple model connecting *ARR17* (and its putative connection to cytokinin), with floral MADS-box genes and their regulators. Further work on the cytokinin pathway in male and female inflorescences of *Populus* spp. and in particular the specific functions of response regulator *ARR17*, will doubtless shed light on this.

## Methods

### Collection of reproductive buds and catkins

Fifteen-year old male and a female *P. balsamifera* trees were chosen in a wild population at Indian Head (50.52°N 103.68°W; elevation 605 m), Saskatchewan, Canada for sampling in summer 2017 and spring 2018. One individual was chosen per sex and consistently sampled throughout the course of the year. Reproductive buds were collected as soon as they were identifiable as such (21 June, summer solstice). Subsequent sampling points were: 21 July, 21 August, 21 September (fall equinox), 21 October, 21 March (spring equinox) and 4 May (emerging catkins). No buds were collected during the dormant period (November to February) as temperature routinely drop to below −20 °C at the Indian Head site.

For each sampling time point, three lateral branches bearing reproductive buds were cut from the trees, placed in water and brought into the laboratory. Five or six developing inflorescences were then squeezed from the bud scales and placed in vials on dry ice. Care was taken not to transfer any resin from the bud scales to the tubes. The vials were then stored at −80 °C until RNA extraction. For each time point four to five developing inflorescences per sex were pooled and subjected to RNA extraction.

Photographs of buds were taken immediately after collection (Canon 80D, macro lens, white backdrop) except for the first stage (June) which was photographed during sample processing.

### RNA extraction and sequencing

Tests were made to compare extraction using CTAB buffer, a silica column-based kit, and Purelink Reagent using manufacturer’s instructions. We found higher yields and better quality using a CTAB-based protocol^40^, which was used for all further work. Turbo DNA-free kit (Thermo Fisher Scientific, MA, USA) was used to remove potential genomic DNA contamination. The resulting samples were checked for quality and consistency by gel electrophoresis and by using an Agilent Bioanalyzer. Depletion of rRNA and library preparation was carried out using the rRNA depleted plant library prep kit (New England Biolabs, MA, US). Qualitatively and quantitatively verified libraries representing four to six catkins were then subjected to paired-end sequencing on a HiSeq 4000 (Illumina) to generate 2×100 bp reads using the Genome Québec platform at McGill University, Canada. This produced 55936031 ± 5106078 (mean ± SD) read pairs per sequencing library and a total of 783104438 read pairs for the experiment (Table S5).

### Sequence processing and analytical methods

Quality and adapter trimmed reads^41^ were mapped to the *P. trichocarpa* reference genome (v3.0, http://phytozome.net) with TopHat v2.1.1 and Bowtie2 v2.3.3 using default parameters and –i 20^42,43^. *P. balsamifera* and *P. trichocarpa* are very closely related and sequencing reads robustly map to the *P. trichocarpa* genome^44^. A summary of sequencing and mapping details is given in Table S5.

Read counts for each gene were obtained with htseq-count using the sorted alignment files as input^45^. Differential transcript abundance was determined based on count data using the edgeR package version 3.26 in R^46^. Applying a weak intensity filter of at least 0.1 cpm (counts per million) in at least two samples retained 78% of the annotated *P. trichocarpa* genes which were then included in downstream analyses. The following package functions were used in conjunction with a manually defined sum-to-zero contrast matrix: estimateDisp, glmFit and glmLRT with robust set to TRUE. Differential gene expression (DGE) was determined using gmlFit and likelihood ratio test or the extactTest functionality of the edgeR package.

To consider variation when using the extactTest, a relatively broad biological coefficient of variation (BCV) value of 0.4 was used^46^. A false discovery rate (FDR) < 0.05 using the Benjamini-Hochberg algorithm was considered for statistical significance. For sex-differences at individual time points, an intensity cut-off for log fold changes of 2 was applied in addition. Gene enrichment analyses were then performed for genes with significant expression difference between male and female samples at each individual time point. The *Arabidopsis* equivalents of poplar genes were used. Gene enrichment analyses were done using the R package gProfileR with g:SCS (Set Counts and Sizes) for multiple testing correction and the genome as background^47^.

Using the controlled functional Gene Ontology vocabulary, detailed information for the category ‘covalent chromatin modification’ (GO:0016569) was downloaded from the AMIGO database (http://amigo.geneontology.org/amigo, as of July 2019, *Arabidopsis* annotation). Transcript abundance data for all expressed poplar homologs of the 247 *Arabidopsis* genes in this category were subjected to hierarchical clustering. Based on general patterns and within-cluster sum of squares, the dendrogram was cut at 0.65. Line plots for major individual subclusters where then generated with the function matplot. Recent literature was used to manually curate further gene function for gene in the major subclusters (see text in results section for details).

Metric multidimensional scaling (MDS) analyses were conducted using the cmdscale function and the plotMDS function of the ‘edgeR’ package. Heat maps were generated using the R packages gplots and ComplexHeatmap^48,49^.

## Supplementary information

Supplementary information.

Supplementary information2.

## Data Availability

All raw sequencing data are available on NCBI’s Sequence Read Archive (http://www.ncbi.nlm.nih.gov/sra); accession number PRJNA578548.
