# Supplementary material for: Gene expression trajectories during male and female reproductive development in balsam poplar (Populus balsamifera L.)
Source: Sci Rep. 2020 May 21;10:8413. doi: 10.1038/s41598-020-64938-w (PMC7242425; doi:10.1038/s41598-020-64938-w)

**Supporting Information**

**Gene expression trajectories during male and female reproductive development in poplar (*Populus balsamifera* L.)**

Quentin Cronk, Raju Soolanayakanahally, Katharina Bräutigam

^1^ Department of Botany, University of British Columbia, Vancouver BC, V6T 1Z4, Canada

^2^ Indian Head Research Farm, Agriculture and Agri-Food Canada, Indian Head, SK, S0G 2K0, Canada

^3^ Department of Biology, University of Toronto, Mississauga ON, L5L 1C6, Canada

**Figure S1.** Rate of gene expression change between successive sampling points increases over time. Here sex is not considered, i.e. and for each time point, month-to-month contrasts include both, male and female sample. Red is upregulation over time, green is downregulation. Little month-to-month change in expression occurs over the first few time points, but this rapidly increases as more complex organs develop which have gene expression patterns specific to particular developmental time point. The final time point has a large amount of specific gene expression associated with pollen formation and flowering.

**Table S1.** Group membership to major clusters and detailed annotation of genes with functions related to 'Covalent Chromatin Modifications'- GO:0016569.

**Table S2.** Genes with largely consistent time-independent sex differences in transcript abundance (main effect of sex).

**Table S3.** All over-represented GO categories among genes with sex differences in expression, analyzed for each developmental stage individually. The g:SCS algorithm for multiple testing correction was used.

**Table S4.** Genes with sex-differential transcript abundance is given for each developmental stage.

**Table S5**. Overview of sequencing information and mapping rates for RNAseq data from male and female samples during the course of reproductiv development in *Populus balsamifera.*

**Figure S1.** Rate of gene expression change between successive sampling points increases over time. Here sex is not considered, i.e. and for each time point, month-to-month contrasts include both, male and female sample. Red is upregulation over time, green is downregulation. Little month-to-month change in expression occurs over the first few time points, but this rapidly increases as more complex organs develop which have gene expression patterns specific to particular developmental time point. The final time point has a large amount of specific gene expression associated with pollen formation and flowering.


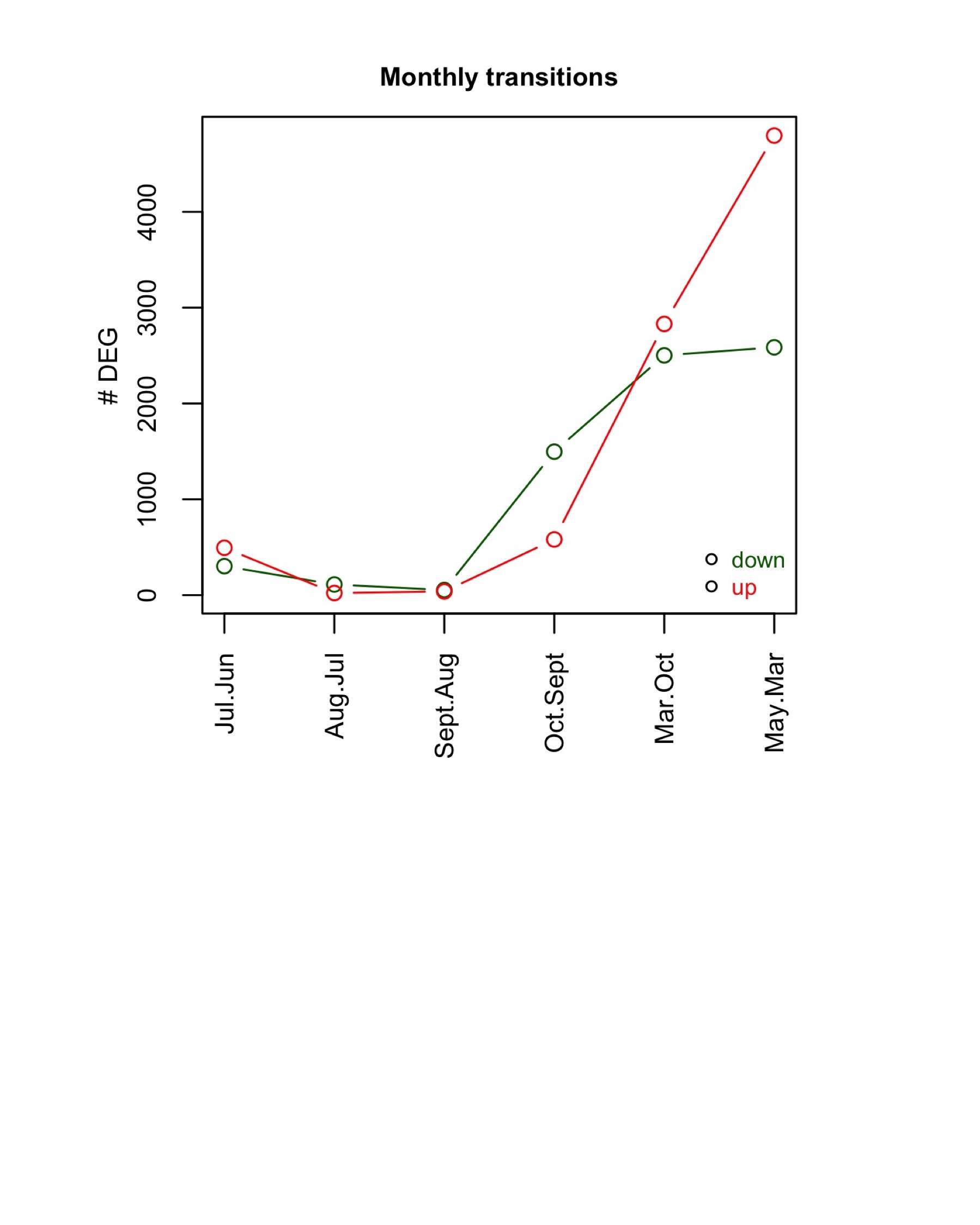

Supplement: Supplementary file 1 — Supplementary information. [file 41598_2020_64938_MOESM1_ESM.docx]
